# Supplementary material for: Unraveling Hematotoxicity of α-Amanitin in Cultured Hematopoietic Cells
Source: Toxins (Basel). 2024 Jan 22;16(1):61. doi: 10.3390/toxins16010061 (PMC10820516; doi:10.3390/toxins16010061)
Supplement: Supplementary file 1 [file toxins-16-00061-s001.zip › toxins-2807936-supplementary.pdf]

toxins

Supplementary Materials: Unraveling Hematotoxicity of α-Amanitin in Cultured Hematopoietic Cells

Willemien F.J. Hof, Miranda Visser, Joyce J. de Jong, Marian N. Rajasekar, Jan Jacob Schuringa, Inge A.M. de Graaf, Daan J. Touw and Bart G.J. Dekkers

Supplementary Materials & Methods

Materials

The hematopoietic cells lines that were used, were HL60 (promyelocytic leukaemia), SUDHL6 (diffuse histiocytic lymphoma) and THP1 (acute monocytic leukaemia) cells, MV411 (biphenotypic B-myelomonocytic leukaemia), K562 (erythroleukemia) cells and Jurkat (T-cell leukemia) cells. All cell lines and primary human CD34+ stem cells were a kind gift of the department of Experimental Hematology, UMCG. Dulbecco's Modified Eagle Medium (DMEM), penicillin-streptomycin (PenStrep), Roswell Park Memorial Institute medium (RPMI), gentamycin, phosphate buffered saline were purchased from Gibco. Foetal bovine serum (FBS), α-amanitin, β-amanitin, Trypan Blue Solution, N-acetylcysteine, silibinin were purchased from Sigma-Aldrich. At Promega, CellTiter 96® AQueous One Solution Reagent for the MTS assay and Caspase-Glo 3/7 assay kit were purchased. The pan-caspase inhibitor Z-VAD(OH)-FMK was from Selleck Chemicals. Benzylpenicillin was purchased at Sandoz, rifampicin at Alsachim and cyclosporin at Cerilliant. At Biolegend, the FITC Annexin V Apoptosis Detection Kit with propidium iodide was purchased. Components for RIPA lysis buffer (50 mM Tris-HCl pH 8.0, 150 mM sodium chloride, 1% Igepal Ca630, 0.5% sodium deoxycholate, 1% sodium dodecyl sulphate), SDS precasted polyacrylamide gels and nitrocellulose membranes were obtained from Bio-Rad. From Thermo Fisher, Protease inhibitor cocktail was purchased. Primary antibody (cleaved caspase 3, rabbit antibody, 0.02% antibody in 3% BSA in TBST solution) was from Cell Signaling Technology. The secondary antibody (GARP 74 polyclonal goat anti-rabbit immunoglobulins/HRP 0.30 g/L, 0.02% antibody in 3% BSA in TBST solution) was from Dako. Western Lightning Ultra mixture was purchased from Perkin Elmer. All other chemicals were of analytical grade. The NovoCyte Quanteon flow cytometer was from Agilent (Santa Clara, CA, USA). NovoExpress software version 1.6.1 was used for analysis.

Cell culture

HL60 (promyelocytic leukaemia), SUDHL6 (diffuse histiocytic lymphoma) and THP1 (acute monocytic leukaemia) cells were cultured in Dulbecco's Modified Eagle Medium (DMEM) with 20% foetal bovine serum (FBS) and 1% penicillin-streptomycin (PenStrep). MV411 (biphenotypic B-myelomonocytic leukaemia) and K562 (erythroleukemia) cells were cultured in DMEM with 10% FBS and 1% PenStrep. Jurkat (T-cell leukemia) cells were cultured in Roswell Park Memorial Institute medium (RPMI) with 10% FBS and 1% PenStrep. HL60 medium also contained 1% gentamycin for trypan blue assay and caspase-Glo assay. All cell lines were cultured at 5% CO2, 37°C and split into fresh medium every 2-3 days, with the exception of THP1 cells being split into fresh medium every 2-3 weeks. CD34+ stem cells were plated in CFC-mix (methocult H4230 medium with 19% Iscove's Modified Dulbecco's Medium (IMDM)), 1% PenStrep, and Interleukin-3 (IL-3), IL-6, stem cell factor (SCF), granulocyte colony-stimulating factor (G-CSF, 20 ng/mL all) and erythropoietin (1 U/mL). This mix was also kindly gifted by the Department of Hematology, UMCG.

Cell number and viability

Effects on cell viability were studied by trypan blue exclusion and MTS conversion assays. Assays were performed according to the manufacturer's instructions. For the trypan blue exclusion assay, HL60 cells plated in duplicate or triplicate on 6-wells plates (200,000 cells/well) for 72 hours in the absence and presence of α-amanitin (0-10 μM). After 72 hours, the cells were washed and incubated with Trypan Blue Solution (0.4%) in PBS. Cells were counted using a Bürker-Türk hemocytometer.

Similarly, MTS (3-(4,5-dimethylthiazol-2-yl)-5(3-carboxymethoxyphenyl)-2-(4-sulfopheny)-2H-tetrazolim) conversion was determined after 72 hours using CellTiter 96® AQueous One Solution Reagent. To this aim, HL60, SUDHL6, THP1, Jurkat cells (20,000 cells/well), MV411 cells (10,000 cells/well) and K562 cells (5000 cells/well) were incubated in transparent 96-wells plates for 72 hours in the absence and presence of α-amanitin or β-amanitin (0-10 μM, both). MV411, K562 and Jurkat cells were plated in quadruplicate, HL60 in quadruplicate or triplicate, SUDHL6 cells in triplicate and THP1 cells in duplicate. HL60 cells that were exposed to α-amanitin for 4 hours or 16 hours, were washed with PBS after 4 hours and 16 hours, respectively, and incubated in the absence of α-amanitin for the remainder of the 72 hours. The corresponding controls were washed simultaneously and re-exposed to an equal concentration of α-amanitin. When applied, the pan-caspase inhibitor Z-VAD(OH)-FMK (100 μM), N-acetylcysteine (1 mM), silibinin (30 μM), benzylpenicillin (1 mM), rifampicin (10 μM) and/or cyclosporin (3 μM) were present during the entire incubation period. When applied, cells were pre-incubated for 30 minutes (with N-acetylcysteine, silibinin, benzylpenicillin, rifampicin or cyclosporin) or 45 minutes (with pan-caspase inhibitor) before 72 hours of exposure to α-amanitin. Next, 20 μL of CellTiter 96® AQueous One Solution was added to each well and the cells were incubated for another 2 hours. Conversion of MTS into its reduced form by mitochondrial cytochromes was measured at 490 nm.

Timelines hit-and-run experiments

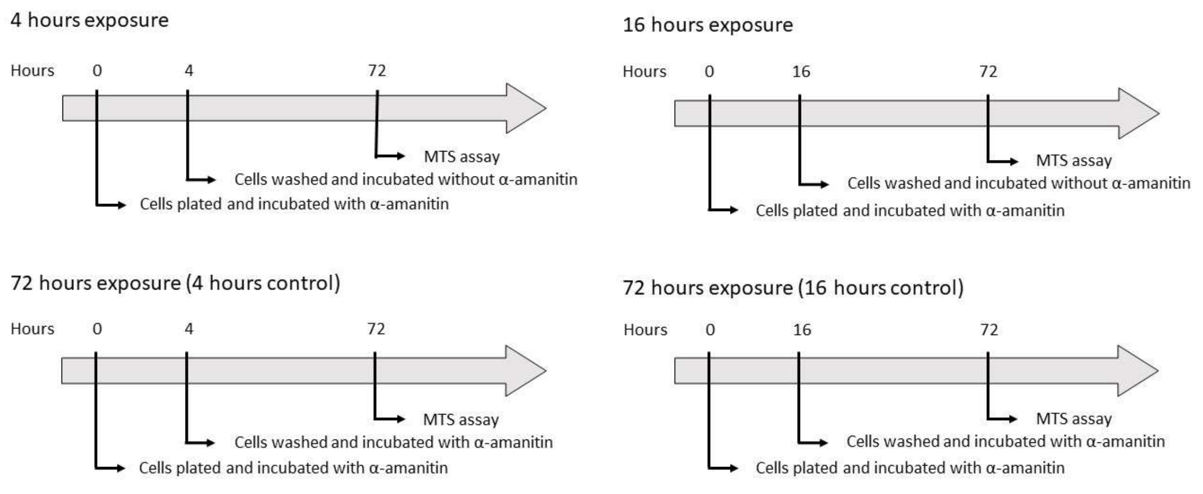

Figure S1: Timelines of the protocols used for the hit-and-run experiments.

Annexin V and PI analysis

The staining solution was freshly prepared by mixing FITC Annexin V, Propidium Iodide (PI) Solution, Annexin V Binding Buffer in ultrapure water in a ratio of 1:1:32:46. Throughout the entire staining, this solution was protected from light. Plated cells were centrifuged at 350 G for 5 minutes and washed once with cold phosphate buffered saline (PBS, pH 7.4). Cells were suspended in PBS at room temperature with staining solution. Final concentrations of FITC-Annexin V and PI were 0.28 μg/mL and 1.6 μg/mL, respectively. The stained samples were analysed by flow cytometry. Cells were excited by a 488 nm laser; detection was performed within 505-560 nm (FITC-Annexin V) and within 595-642 nm (PI). Cells were counted and divided into viable, early apoptotic, late apoptotic, and necrotic subpopulations.

Colony-forming cell formation assays

Colony-forming cell (CFC) assays were performed as described earlier [56]. In short, CD34+ stem cells were incubated in 35 mm Petri dishes (500 cells/dish in CFC-mix) in the absence and presence of α-amanitin (0-10 μM) for 14 days. Afterwards, stem cell colonies were counted using the colony-forming unit assay. Subsequently, samples were washed with PBS and total cells were counted using a hemocytometer. The different colonies were distinguished based on morphology.

Caspase-Glo 3/7 assay

The Caspase-Glo 3/7 assay kit was used for detection of caspase activation in HL60 cells according to the manufacturer's instructions. Cells (20,000 cells/well) were plated in triplicate on white 96-well plates for 24 hours in the absence and presence of α-amanitin. Caspase 3/7 reagent (50 μL) was added to each well and the plate was incubated on a rotary shaker at room temperature for 1 hour. Generation of luminescence was measured with the Synergy H4 microplate reader.

Preparation of protein samples

HL60 cells were incubated for 24 hours on 6-wells plates (1,000,000 cells/well) in the absence and presence of α-amanitin (0-10 μM). Cells were washed with cold PBS and centrifuged (300G, 5 minutes). Protein was isolated by adding RIPA lysis buffer, 0.4% Protease inhibitor cocktail, 0.1% 100 mM sodium orthovanadate, 0.1% 1 M sodium fluoride and 10 mM β-mercapto-ethanol). Samples with protein concentration of 200 μg/100 μL were boiled at 100 °C for 5 min.

Western blot analysis

Cleaved caspase-3 was quantified by Western blot analysis. Equal amounts of protein were separated on 4-20% SDS precasted polyacrylamide gels and transferred to a nitrocellulose membrane. To avoid non-specific binding, the membrane was blocked in 5% w/v skimmed milk in TBST (1.5 M NaCl, 0.1 M Tris pH 8.0 and 0.04% Tween 20 in H_2O) for 20 minutes. Primary antibody incubation was performed overnight at 4 °C. After washing the membrane 3 times with TBST for 5 min, incubation with the secondary antibody was performed for 1 hour at room temperature. Western Lightning Ultra mixture was added to the membrane to detect the proteins, which were visualized in the ChemiDoc MP Imaging System. All bands were normalized for total protein and normalized for 10 μM α-amanitin.

Supplementary results

Table S1: Statistical evaluation of IC50 values

Table S1: IC50 values with statistically significant differences. Data represent mean ± SD of 3-6 experiments; *p<0.05, **p<0.01, ***p<0.001.

Cell line	IC_{50} (µM)	MV411	THP1	CD34+ cells	Jurkat	CD34+ colonies	K562	SUDHL6	HL60
MV411	0.59±0.07	-	-	-	-	-	*	***	***
THP1	0.72±0.09	-	-	-	-	-	-	***	***
CD34+ cells	0.72±0.50	-	-	-	-	-	-	***	***
Jurkat	0.75±0.08	-	-	-	-	-	-	***	***
CD34+ colonies	1.03±0.58	-	-	-	-	-	-	***	***
K562	1.95±0.18	*	-	-	-	-	-	**	***
SUDHL6	3.61±1.02	***	***	***	***	***	**	-	-
HL60	4.49±0.73	***	***	***	***	***	**	-	-

Effect of silibinin on α-amanitin-induced toxicity in HL60 cells

As silibinin was found to decrease MTS reduction in presence of α-amanitin 3 µM, a full α-amanitin concentration response curve was made in the absence and presence of silibinin (30 µM, Figure 6). Silibinin had a slight toxic effect by itself, evident by the small decrease in MTS reduction in absence of α-amanitin. However, this did not reach statistical significance. The combination of silibinin and α-amanitin appeared to increase the effect of α-amanitin alone. At 0.1 and 0.3 µM, α-amanitin in combination with silibinin significantly decreased MTS reduction relative to the control. The largest effect of silibinin was found when combined with α-amanitin 3 µM. Silibinin significantly enhanced the effect of α-amanitin 3 µM on MTS reduction (p<0.001), resembling the effect of 10 µM α-amanitin. In line, a significantly lower IC_{50} was found for α-amanitin with silibinin (2.03±0.04 µM) than for α-amanitin alone (4.18±0.68 µM, p<0.05).

Silibinin induces toxicity of α-amanitin in HL60 cells

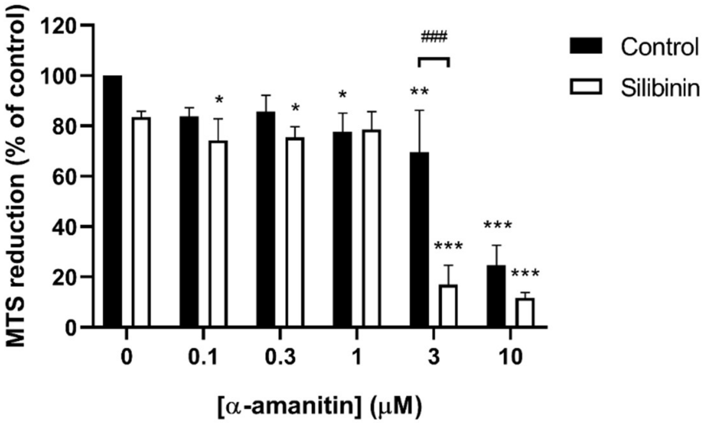

Figure S2: Silibinin increases α-amanitin-induced toxicity in a concentration-dependent manner in HL60 cells. Data represent mean ± SD of 3 experiments. *p<0.05, **p<0.01, ***p<0.001 compared to control (0 µM α-amanitin without silibinin), ###p<0.001 compared to equal concentration α-amanitin.

α-Amanitin significantly reduces cell viability and induces apoptosis and necrosis in a time-dependent and concentration-dependent manner

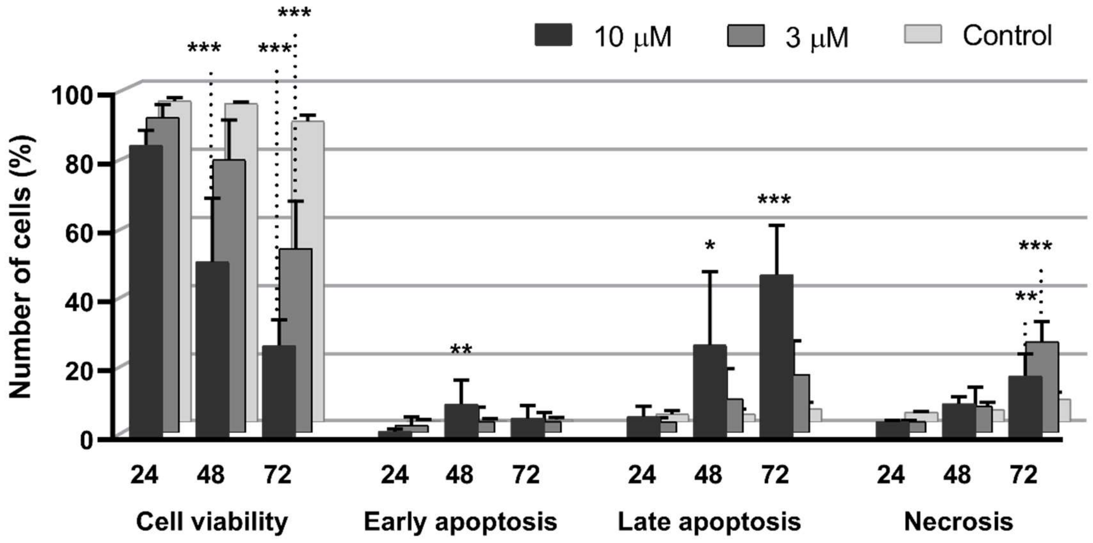

Figure S3: α-Amanitin decreases the percentage of viable cells and increases the percentage of apoptotic and necrotic cells. Annexin V/PI viability analysis of HL60 cells after 24, 48 and 72 hours of incubation with α-amanitin. Bars represent mean ± SD of 3 experiments. *p<0.05, **p<0.01, ***p<0.001 compared to control (0 µM α-amanitin).

Hit-and-run effect of α-amanitin

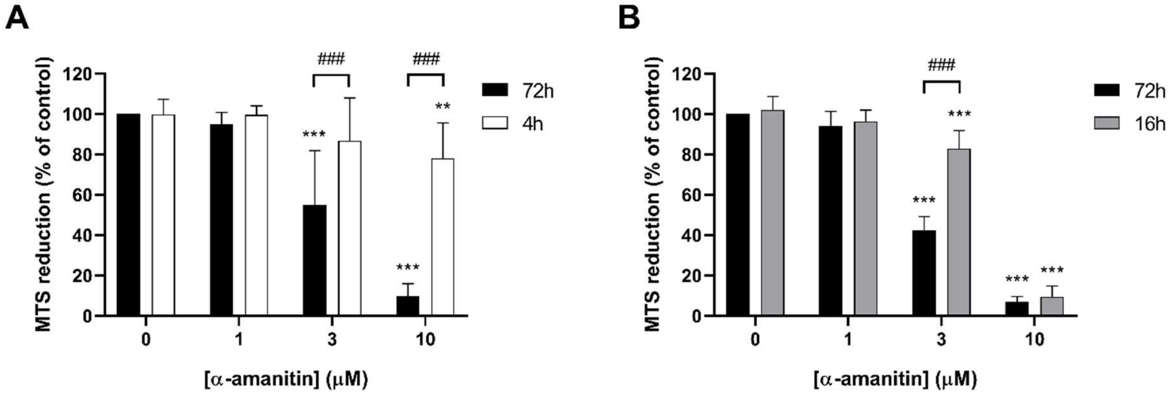

Figure S4: Hit-and-run exposure to α-amanitin during 16 hours reduces viability at 72 hours. Cells were exposed to α-amanitin (0-10 μM) for [a] 4 hours and [b] 16 hours after which medium was replaced by medium without α-amanitin for the remainder of the 72 hours. Bars represent mean ± SD of 4-6 experiments. **p<0.01, ***p<0.001 compared to control (0 μM α-amanitin, 72 hours of exposure), ###p<0.001 compared to 72 hours of exposure to equal concentration α-amanitin.
